# Developmental Changes in Mismatch Responses to Mandarin Consonants and Lexical Tones from Early to Middle Childhood

**DOI:** 10.1371/journal.pone.0095587

**Published:** 2014-04-22

**Authors:** Huei-Mei Liu, Yuchun Chen, Feng-Ming Tsao

**Affiliations:** 1 Department of Special Education, National Taiwan Normal University, Taipei, Taiwan; 2 Department of Psychology, National Taiwan University, Taipei, Taiwan; UNLV, United States of America

## Abstract

The purpose of this study was to use mismatch responses (MMRs) to explore the dynamic changes of Mandarin speech perception abilities from early to middle childhood. Twenty preschoolers, 18 school-aged children, and 26 adults participated in this study. Two sets of synthesized speech stimuli varying in Mandarin consonant (alveolo-palatal affricate vs. fricative) and lexical tone features (rising vs. contour tone) were used to examine the developmental course of speech perception abilities. The results indicated that only the adult group demonstrated typical early mismatch negativity (MMN) responses, suggesting that the ability to discriminate specific speech cues in Mandarin consonant and lexical tone is a continuing process in preschool- and school-aged children. Additionally, distinct MMR patterns provided evidence indicating diverse developmental courses to different speech characteristics. By incorporating data from the two speech conditions, we propose using MMR profiles consisting of mismatch negativity (MMN), positive mismatch response (p-MMR), and late discriminative negativity (LDN) as possible brain indices to investigate speech perception development.

## Introduction

Studying speech perception in childhood can improve the understanding of how humans learn to process speech sounds and how people become culture-bounded and attend to the specific sounds in their native languages. Numerous discoveries have been made regarding infant perception since Eimas et al. [1] conducted their seminal study. Additionally, the difference in the speech-related perception abilities in infants may predict their language performances in the early childhood [2]–[4]. However, few studies have focused on the speech perception of typically developing children older than 1 or 2 years of age.

Examining the developmental course of speech perception abilities in childhood is theoretically critical for several reasons. First, the substantial vocabulary increase during early childhood produces changes in the manner in which speech patterns are represented and processed. Second, during middle childhood, when children learn to read, the writing system might affect phonological representations and processing. Finally, because children are continually exposed to their native languages, perception is expected to be refined and altered before they reach a stable adult-like status. Some studies have suggested that the speech categorization, discrimination, and recognition abilities in preschool and school-aged children are not as mature as those of adults [5]–[8]. Children have also been found to be incapable of using the fine-grained spectral cues under adult-directed speech as well as adults do in discrimination and identification tasks [9]–[10]. Nevertheless, other studies have failed to observe age variations in identification tasks among children from grade 2 to grade 5 and in speech discrimination tasks among school-aged children, teenagers, and adults [11]–[12]. While most studies were conducted in children with non-tonal languages, only limited studies investigated speech perception in young children with tonal languages such as Mandarin Chinese and Cantonese [13]–[15]. More research examining the general course of speech perception development in children across languages could help to explore if the linguistic background predisposes to different developmental trajectories in children.

Previous studies have reported that children's performance is influenced by various speech discrimination tasks [16]–[17]. In addition, behavioral tasks require children's overt responses, and thus, are often influenced by other cognitive factors, including motivation, motor response abilities, and individual response strategies. Therefore, the use of behavioral tasks might not accurately reflect the speech perception abilities in children. To circumvent the problem caused by behavioral tasks, researchers studying speech perception have focused on using auditory event-related potentials (ERPs) as a relatively objective electrophysiological measure for mirroring the brain's responses to discriminate speech features. One of the ERP components is mismatch negativity (MMN), which is elicited by an auditory passive oddball paradigm containing rare (deviant) stimuli presented in the sequence of frequent (standard) stimuli. MMN is quantified by subtracting the averaged waveform to standard stimuli from the averaged deviant waveform, and usually peaks between 100 to 250 ms from stimulus change onset [18]. The MMN amplitude is associated with the behavioral discrimination performance of between-sound differences [19]–[21]. Children also exhibited enhanced MMN amplitude in discriminating between specific speech sounds after short-term training [22]–[23], suggesting that MMN could be used as an index of speech perception sensitivity. Apart from MMN, a second negativity has been elicited by deviant stimuli in the auditory passive oddball experiment. This negativity was first reported in children by Korpilahti et al. [24] and has been referred to as late MMN (lMMN) or late discriminative negativity (LDN) in relevant studies. This negative component appears after MMN and peaks at approximately 400–430 ms in response to changes in speech stimuli for both children and young adults [25]–[27]. Compared with MMN, LDN was stably observed in children aged 2 to 3 years to changes in fundamental frequency, duration, intensity, and source in complex tones; thus, researchers suggested LDN to be a candidate for the early index of auditory learning and development [28]. The converging patterns of MMN and LDN are suitable for studying speech perception development in childhood, particularly in situations where behavioral responses cannot be readily elicited.

Research using MMN to explore the developmental trajectory of preschool to school-aged children in perceiving distinct speech sounds is scarce. Kraus et al. [27] recorded the MMN responses to the standard /ga /and deviant /da/ of 7- to 11-year-old children and adults. The results indicated no variation in peak latency and the duration of MMN between the child and adult groups. Unexpectedly, the mean amplitude of MMN was larger in the child group than in the adult group. McGee et al. [29] reported that school-aged children yielded significant MMN responses to two sets of consonant contrast: /da/−/ga/ and /wa/−/ba/, even when the specific acoustic feature was manipulated to a relatively difficult level (i.e., small difference in F3 onset frequency and formant transition duration between /da/−/ga/ and /wa/−/ba/, respectively). Regarding younger children (6 to 7 years), Maurer, Bucher, Brem, and Brandeis [30] included a standard /ba/ and deviants varying in acoustic differences to standard (large deviant /ta/ and small deviant /da/) in a two-deviant oddball paradigm. The results from the low-resolution electromagnetic tomography (LORETA) indicated a consistent frontal positive mismatch response (p-MMR) with posterior negativity in children, whereas adults exhibited a frontocentral MMN with mastoid positivity. The researchers argued that the age-appropriate frontal positive mismatch responses reflected automatic mismatch detection, and the developmental trajectory must involve a reduction of this large frontocentral positive mismatch response for MMN in adults to emerge.

Instead of using consonant-vowel (CV) syllables, Čeponienė et al. [25] examined the sensitivity to vowels in a group of 3-year-old children. The stimuli were the standard vowel /a/, one across-category vowel deviant /o/, and one within-category nasalized vowel /ã/. Their results indicated a modulation of phonetic category on MMN. In other words, the response to the across-category vowel was larger in amplitude, indicating that the vowel discrimination in 3-year-old children is as sensitive to the phonemic aspects of stimulus change as is that in adults. Compared with results from other studies, the vowel-elicited MMN peaked considerably later in 3-year-old children than in school-aged children [31]–[32], indicating that 3-year-old children require a longer processing time than older children do in discriminating sounds. Recently, Shafer et al. [33] examined the maturation of the MMN response to an English vowel contrast/i/−/e/ in children between the ages of 4 and 7 years. The results indicated that the older group (6–7 years) exhibited two negative peaks between 100 and 400 ms. Regarding the younger children (4–5 years), a large positivity peak between 100 and 250 ms occurred before the negative response. Based on the results, the researchers proposed that the maturation of vowel discrimination can be indexed according to the early peak latency of the negative component from 300 to 400 ms and the disappearance of the p-MMR. In summary, various developmental indices of MMRs have been identified, including changes in amplitude, latency, or polarity from early to middle childhood. Because the maturation timetable for the changes in MMRs may be feature specific, the core developmental characteristic should be determined by detailing the development of mismatch responses to various specified speech features in each language.

Regarding a tonal language such as Mandarin, studies on the developmental trajectories of MMN typically address consonant, vowel, and lexical tone. A Mandarin syllable may be composed of four possible elements: Tone and vowel serve as the compulsory units, and consonants are optional units occurring in either the initial or final position. Among these units, tone has been considered to be the most salient because of its compulsory status for every syllable [34]. Mandarin Chinese has four tones: ma1 ‘mother’ [T1], ma2 ‘hemp’ [T2], ma3 ‘horse’ [T3], ma4 ‘scold’ [T4]. Tones 1 to 4 can be described phonetically as high level, high rising, low falling rising, and high falling, respectively [35]. The dominant acoustic cue for lexical tone is the change in pitch contour [36], and discrimination between different tone pairs follows diverse developmental courses. Among four tones, the pitch contour for Tone 2 [T2] is acoustically similar to that of Tone 3 [T3], and data derived from perception and production tasks have confirmed that T2 and T3 is the most confusing tone pair for children and second language learners [15], [37]–[38]. In addition to lexical tones, various types of consonants occur, and most of the consonant pairs differ only in subtle acoustic cues. Therefore, although the consonant is not a compulsory unit in a Mandarin syllable, the ability to discriminate subtle acoustic cues carried by different consonants is crucial for children in order to develop delicate phonological representation. The discrimination performance for consonants with high discriminative difficulty (e.g., retroflex fricative-affricate contrasts) in school-aged children has not yet reached the adult level [39]. Adults' discrimination of the affricate-fricative consonant also has not yet reached the error-free level (mean = 95.21%) [40]. Thus, studies exploring speech perception abilities in children of various ages that include lexical tone and consonants with high discriminative difficulty can reflect the developmental stages of different speech characteristics.

According to our review of relevant research, few studies have been conducted on MMN response in Mandarin-speaking children. Meng et al. [41] tested MMN response in 8- to 13-year-old dyslexic and nondyslexic children. The speech stimuli contrasts were initial consonants (/da/−/ga/), rhyming part (/dan/−/dai/), and lexical tone (/ba1/−/ba2/). Differences between the two groups were observed in MMNs to syllables deviating in initial consonants and vowels, but no group difference in lexical tone condition were observed. Nevertheless, because of the lack of original waveform and amplitude measures for the typically developing group in the study by Meng et al, interpreting the development patterns seems difficult based on the results. Zhang et al. [42] used the /pa2/-/pa4/ continuum to examine the categorical perception of Mandarin lexical tone in school children with or without dyslexia. The results indicated that the age-matched control group demonstrated a significantly enhanced MMN to the across-category deviants compared with the within-category deviants, whereas the dyslexic children did not exhibit such effects. The enhanced MMN elicited by across-category tonal contrast indicated that 10-year-old typically developing Mandarin-speaking children have formed phonological representations of lexical tones similar to those of adults. Lee et al. [43]measured the MMN to Mandarin lexical tones, initial consonants, and vowels in 4- to 6-year-old preschoolers by using the two-deviant oddball paradigm to investigate the effects of age, phonological saliency, and deviance size on the presence of MMN and p-MMR. The results indicated that, for the compulsory elements of Mandarin syllables (i.e., lexical tones and vowels), the larger deviants elicited adult-like MMNs and the smaller deviants elicited p-MMRs. The optional elements of the Mandarin syllables (i.e., the initial consonant) only elicited p-MMR in the preschoolers. The results may indicate that the transition from p-MMR to adult-like MMN provides information on whether speech perception in children becomes mature and automatic.

In summary, behavior and electrophysiological studies exploring speech perception development in children are limited, and the clear developmental trajectory remains unknown. Therefore, the main purpose of the present study was to use MMRs profiles, consisting of MMN, p-MMR and LDN, as possible brain indices to explore the dynamic change in Mandarin speech perception from early to middle childhood. Compared with previous studies, we included two children groups exhibiting a wider age range to examine clearer developmental patterns. In addition, we also included an adult group as the maturation status for comparison. The advantage of using an MMN paradigm is that possible cognitive factors caused by the wide age range can be reduced. We also used synthetic consonant and lexical tone pairs varying only in specific acoustic features that serve as crucial perceptual cues for discriminating the speech contrasts in Mandarin Chinese. By using brain responses as sensitive measures, we anticipated to observe varying developmental time courses for children's abilities to capture specific speech cues. Finally, the developmental change pattern in MMRs, such as that in latency, polarity, and topography, is not yet clear or complete because of children's varying ages and the selection of speech stimuli. The results of using two speech sounds and wider age groups in the same study might assist in establishing the developmental trajectories of the MMRs.

## Methods

### Participants

A total of 26 native monolingual Mandarin-speaking adults (10 women, mean age  = 22.4 years, SD = 3.27), 20 preschool children (7 girls, mean age  = 3.40 years, SD = 0.46), and 18 school-aged children (8 girls, mean age  = 8.57 years, SD = 0.54) participated in this study. The participants were recruited from a metropolitan area through advertisements. All of the participants exhibited normal visual acuity and hearing acuity, and had no history of neurological diseases according to self- and parental reports. Children were screened for normal language development using standardized tests (preschool: Child Language Disorder Scale-Revised; school: Peabody Picture Vocabulary Test-Revised) [44]–[45] and all of the children scored above -1SD of mean on the tests, indicating that their language ability was at the age-appropriate level. The research protocol was reviewed and approved by Institutional Review Board of National Taiwan Normal University. Informed consent forms signed by all of the adult participants and parents of the child participants were collected before experiment is conducted.

### Stimuli and Design

**Figure 1:**
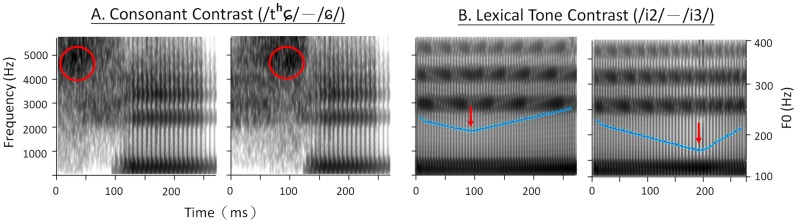
Spectrograms of Mandarin speech stimuli used in the study. (A) The *consonant* differ only in the time at which maximum amplitude is reached during the initial frication portion (marked with red circles); (B) The *lexical tone* differ only in the pitch contour as reflected by the turning point of the fundamental frequency (F0) (blue lines and marked with red arrows).

Since our purpose is to delineate the fine-grained developmental trajectory of MMRs across the age groups, we used the behavioral data in choosing the two contrasts which not only be able to show the developmental changes in neural response patterns but also create different levels of discriminative difficulties. Mandarin affricate-fricative contrast (/t_
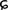
^h^_/-/_
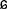
_/) and lexical tone pairs (/i2/-/i3/) from our previous behavioral study examining the speech discrimination abilities of 4- to 8-year-old children were used. The range of mean accurate rate in the discrimination task from 4- to 8-year-old groups were 58.6–71.2% for the consonant contrast and 63.9–88.0% for the lexical tone contrast [46]. Although the acoustic change is subtle for the /i2/-/i3/ pair, some of the 8-year-old children could discriminate this contrast to 100% level (mean = 88%); so this contrast was taken as an easy control in this study. Based on the behavioral data, the consonant sounds (/t_
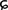
^h^_/-/_
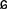
_/) are of higher discriminative difficulty than the lexical tone pair (/i2/-/i3/). All of the stimuli were synthesized using Praat [47] and normalized in RMS intensity. The use of synthetic stimuli is to examine the ability to weight most prominent perceptual cues in discriminating speech contrasts across age groups. The goodness of these speech stimuli in terms of their own native-language phonemes were judged by native Mandarin adult speakers in a pilot study, to ensure the naturalness of the stimuli. Figure 1 shows the spectrograms of the two sets of stimuli, and the acoustic parameters are presented as follows.

#### Consonant

The Mandarin consonant stimuli consisted of CV syllables consisting of an alveolo-palatal affricate (/t_
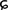
^h^_/, standard) and an alveolo-palatal fricative (/_
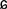
_/, deviant). The two token were selected from a continuum varying in the time point of the maximal rise in amplitude during the initial 130-ms frication portion. The affricate consonant exhibited a short amplitude rising time (maximal amplitude occurring at 30 ms) and the fricative consonant exhibited a long amplitude rising time (maximal amplitude occurring at 100 ms). The two syllables were 275 ms in duration and consisted of an identical steady-state vowel /i/ with formant frequencies of 293, 2,274, 3,186, and 3,755 Hz and bandwidths of 80, 90, 150, and 350 Hz, respectively. The fundamental frequency of the syllable was 120 Hz (high flat tone, Tone 1 in Mandarin).

#### Lexical Tone

The Mandarin lexical tone stimuli consisted of syllables /i2/ with a rising tone (standard) and syllable /i3/ with a low dipping tone (deviant). The two stimuli were from a lexical tone continuum and were identical to each other except for pitch contour, reflected in the variation in fundamental frequency (F0). The acoustic features of F0 in /i2/ were onset  = 219 Hz, turning point (at 34% of the syllable duration)  = 195 Hz, endpoint  = 245 Hz; and those in /i3/ were onset  = 216 Hz, turning point (at 71% of the syllable duration)  = 156 Hz, endpoint  = 209 Hz. The total duration of the syllables was 270 ms. The formant frequencies of the steady-state vowel /i/ were 290, 2,815, 3,945, 4,973 Hz and the bandwidths were 100, 220, 115, 239 Hz, respectively.

### Procedure

Two oddball blocks consisting of consonant and lexical tone contrasts were presented to each participant in a counterbalanced sequence among the participants. The ratio of standard to deviant was 8∶1, and the total number of stimuli in each presentation block was 1,080. The presentation of stimuli was in a pseudo randomized order with at least three successive standards between deviants. The interstimulus interval (ISI) was 610 ms for the adults and school children. To reduce the recording time for preschoolers, the ISI was set to 430 ms because no ISI effect on the amplitude of MMN within this range was reported [48]. Speech stimuli were presented over loudspeakers at 70 dB SPL. The participants were seated in a comfortable chair in a sound-attenuated electrically shielded room and were instructed to watch silent self-selected movies, ignore the sound stimuli, and sit as quietly as possible. Regarding the preschooler group, a research assistant and parents accompanied the child in the room and comforted the child when necessary. Breaks were given when requested by the participant.

### EEG Recording and Data Analysis

Electroencephalogram (EEG) was recorded using 32 Ag/AgCl electrodes embedded in an elastic cap (QuickCap, Neuromedical Supplies, Sterling, VA, USA). For the comfort of the preschool children, only six electrodes (F3/Fz/F4, C3/Cz/C4) in the cap were used. The selection of the electrodes was based on previous studies that suggested that MMN is most prominent in frontocentral locations [33], [43]. The vertical electrooculogram (EOG) (VEOG) was monitored using electrodes placed above and below the left eye. The horizontal EOG (HEOG) was recorded using a bipolar montage with two electrodes placed on the right and left external canthus. The continuous EEG signal was amplified with a bandpass from 0.05 to 70 Hz by using the SynAmps2 (Neuroscan, Inc.) amplifier and digitized at a sampling rate of 500 Hz. All electrodes were online referenced to the average of the left and right mastoids. Electrode impedances were maintained below 10 KΩ during the recording. Regarding the offline analysis, eye-blink correction to the raw EEG data was implemented using the linear regression function provided by NeuroScan software [49]. The continuous EEG data were epoched with 100 ms of prestimulus intervals and 600 ms of poststimulus intervals. The prestimulus interval (−100 to 0 ms) was used for baseline correction. Data were lowpass-filtered by 30 Hz (12 dB/octave). Any epoch containing voltage variation that exceeded ±150 µV for the two children group (±90 µV for the adult group) at HEOG and any other electrodes was considered as an artifact and was rejected. Average ERPs were calculated separately for standard and deviant stimuli to consonant and lexical tone contrasts for individual participants, and difference waveforms were created by subtracting the standard waveform from the deviant waveform for each participant. The average number of accepted deviants (SD in parentheses) for the preschool, school, and adult groups were 90 (15), 99 (12), 99 (12) to the consonant contrast, and 91 (12), 101 (10), and 100 (12) to the lexical tone contrast, respectively. The accepted numbers of deviants in this study were within the reasonable range from other related studies working with preschool children [25], [43].

The mean amplitudes of the standard and deviant waveforms for each participant were calculated for the seven successive 50-ms intervals. The 50-ms successive time window analysis was chosen for two main reasons: 1) the MMR patterns may change across age groups, selecting the same time window based on the manipulated acoustic feature in the synthetic stimuli provides the same base for cross-group comparison; and 2) The split time window approach has been used in the related papers investigating the developmental courses in MMRs in preschool-aged children [33], [43], [50], so the same approach may provide the base for comparison with previous literatures. Based on the conventional definition of MMN (i.e., 100 ms after the onset of acoustic difference between the standard and deviant sounds), the starting point of the first time window was 130 ms for consonant and 185 ms for lexical tone.

Since the possible varying pattern of MMRs across groups is the main interest of this study, a four-way ANOVA was first conducted for consonant and lexical tone with the groups (preschool, school, and adult) as a between-subject factor and with condition (standard/deviant), time window (seven 50-ms intervals), and electrodes (F3/Fz/F4/C3/Cz/C4) as within-subject factors. The results of the first level analysis served as the basis for following detailed analyses. If significant interactions related to group were found, further analyses were done for each age group. For each group, repeated-measure ANOVAs with condition (standard/deviant), hemisphere (left, midline, and right), and site (frontal/central) as within-subject factors were conducted in seven successive time intervals for each speech contrast. The purpose of separate ANOVAs for each group is to determine when the standard and deviant began to differ. In addition, the possible developmental MMR effects on topography were also investigated in the separate group analysis by regrouping the six electrodes according to the hemisphere and caudality site domain. Regarding all ANOVAs, the Greenhouse-Geisser adjustment was applied to correct for violations of sphericity associated with repeated measures. Post-hoc pairwise comparisons were performed using the Bonferroni test and only significant differences (*p<*.05) are reported.

## Results

**Figure 2:**
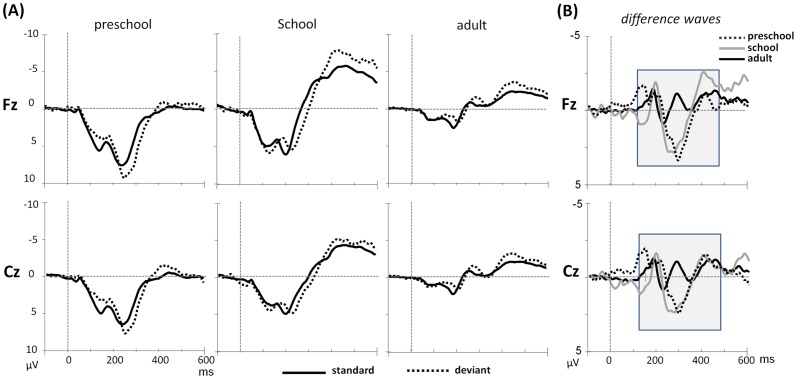
(A) Grand average waveforms of the standard and deviant stimuli at midline electrodes (Fz & Cz), and (B) difference waveforms across three age groups to consonant contrast.

**Figure 3:**
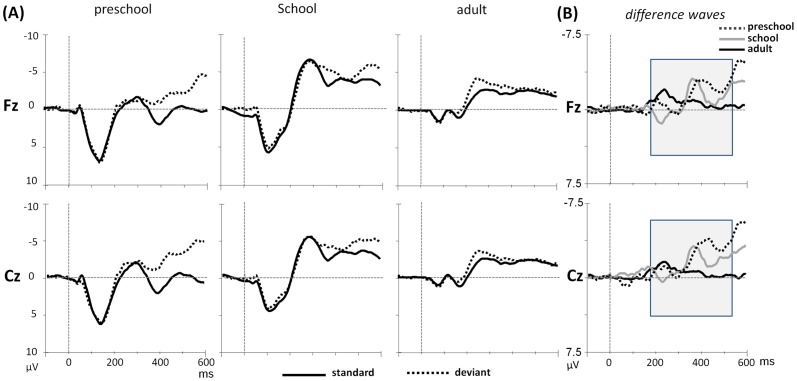
(A) Grand average waveforms of the standard and deviant stimuli at midline electrodes (Fz & Cz), and (B) difference waveforms across three age groups to lexical tone contrast.

The average ERPs for the standard and deviant stimuli at the midline electrodes (Fz & Cz) as well as the difference waveforms across the three age groups to consonant and lexical tone pairs were shown in figure 2 and 3, respectively. The developmental trend was first observed in the general amplitude of the ERP responses in both standard and deviant waveforms. Regarding both speech stimuli, the overall positive amplitude changed to negative in preschool and school-aged children and evidently decreased in the adult group. The decrease in amplitude from the child to adult stage in our data is consistent with that of previous studies [50]. The analyses of MMRs by comparing the standard and deviant waveforms for each speech contrast are discussed below.

### Consonant

The seven time windows selected for analysis were 130 to 180, 180 to 230, 230 to 280, 280 to 330, 330 to 380, 380 to 430, and 430 to 480 ms. The results of the ANOVA revealed the main effects of the time window (*F*(6,366) = 292.44, *p*<.001, η*_p_* ^2^ = .827). Significant two-way interactions between time window and group (*F*(12,366)  = 43.05, *p*<.001, η*_p_* ^2^ = .585), electrode and group (*F*(10,305)  = 6.14, *p*<.001, η*_p_* ^2^ = .168), condition and time window (*F*(6,366)  = 39.79, *p*<.001, η*_p_* ^2^ = .395), and time window and electrode (*F*(30,1830)  = 29.40, *p*<.001, η*_p_* ^2^ = .325) were observed. Significant three-way interactions of condition × time window × group (*F*(12,366)  = 10.55, *p*<.001, η*_p_* ^2^ = .257), time window × electrode × group (*F*(60,1830)  = 6.16, *p*<.001, η*_p_* ^2^ = .168), and condition × time window × electrode (*F*(30,1830)  = 3.66, *p*<.001, η*_p_* ^2^ = .057) were also observed. A four-way interaction of condition × time window × electrode × group (*F*(60,1830)  = 2.16, *p* = .002, η*_p_* ^2^ = .066) was also observed. Because significant interactions related to group were found, further analyses for each group are reported separately to examine the changes in the differences between standard and deviant stimuli (i.e., MMRs) across time windows.

#### Preschool children

Significant main effects of condition were observed in the intervals of 230–280 ms (*F*(1,19)  = 7.24, *p* = .015, η*_p_* ^2^ = .276) and 280–330 ms (*F*(1,19)  = 19.20, *p*<.001, η*_p_* ^2^ = .503). A significant two-way interaction of condition and hemisphere was observed in the interval of 280–330 ms (*F*(2,38)  = 3.77, *p* = .034, η*_p_* ^2^ = .165). A post-hoc comparison revealed that the differences between the standard and deviant stimuli at the right hemisphere were more positive than those at the midline channels (*p* = .009).

#### School children

Significant main effects of condition were observed in the intervals of 230–280 ms (*F*(1,17)  = 25.67, *p*<.001, η*_p_* ^2^ = .602), 280–330 ms (*F*(1,17)  = 11.24, *p* = .004, η*_p_* ^2^ = .398), and 380–430 ms (*F*(1,17)  = 7.41, *p* = .014, η*_p_* ^2^ = .304). A significant two-way interaction between condition and site was observed in the intervals of 280–330 ms (*F*(1,17)  = 4.88, *p* = .041, η*_p_* ^2^ = .223) and 380–430 ms (*F*(1,17)  = 6.31, *p* = .022, η*_p_* ^2^ = .271). Post-hoc comparisons revealed that, compared with the central sites, the deviant-standard difference was more positive at the frontal sites in the intervals of 280–330 ms (*p* = .041) and more negative in the interval of 380–430 ms (*p* = .022). A three-way interaction of condition, hemisphere, and site was observed in the interval of 230–280 ms (*F*(2,34)  = 4.99, *p* = .015, η*_p_* ^2^ = .227), suggesting that the positive difference between standard and deviant stimuli was greatest at the frontal left site (F3) and smallest at the central right site (C4) (*p* = .043).

#### Adult group

Significant main effects of condition were observed in three time windows: 130–180 ms (*F*(1,25)  = 6.38, *p* = .018, η*_p_* ^2^ = .203), 380–430 ms (*F*(1,25)  = 5.25, *p* = .031, η*_p_* ^2^ = .174), and 430–480 ms (*F*(1,25)  = 5.06, *p* = .034, η*_p_* ^2^ = .168). Significant interactions between condition and hemisphere were also observed in these three intervals (*F*(2,50)  = 4.61, 6.71, 6.31; *p* = .023, 003, 004; η*_p_* ^2^ = .156, .212, .201, respectively). A post-hoc comparison showed that the deviant was more negative than the standard at the midline electrodes (*p*<.005).

To summarize the results of the consonant contrast, the three groups exhibited significant main effects of condition in the various time windows. Both the preschool- and school-aged children exhibited positive responses to the deviant stimuli during the interval of 230 to 330 ms after the stimulus onset, but the school-aged children exhibited additional negative responses to the deviant sound in the later time interval of 380 to 430 ms. The typical early MMN response in the time window of 130 to 180 ms can be observed only in the adult group, and the MMN response was most prominent at the midline electrodes.

### Lexical Tone

The seven time windows selected for analysis were 185 to 235, 235 to 285, 285 to 335, 335 to 385, 385 to 435, 435 to 485, and 485 to 535 ms. The results of the ANOVA revealed the main effects on condition (*F*(1,61) = 14.84, *p*<.001, η*_p_* ^2^ = .196), time window (*F*(6,366) = 38.14, *p*<.001, η*_p_* ^2^ = .385), and electrode (*F*(5,305) = 6.37, *p*<.001, η*_p_* ^2^ = .095). Significant two-way interactions between time window and group (*F*(12,366) = 7.34, *p*<.001, η*_p_* ^2^ = .194), electrode and group (*F*(10,305) = 7.44, *p*<.001, η*_p_* ^2^ = .196), condition and time window (*F*(6,366) = 13.44, *p*<.001, η*_p_* ^2^ = .181), and time window and electrode (*F*(30,1830) = 5.63, *p*<.001, η*_p_* ^2^ = .084) were observed. Significant three-way interactions of condition × time window × group (*F*(12,366) = 11.42, *p*<.001, η*_p_* ^2^ = .272) and time window × electrode × group (*F*(60,1830) = 3.41, *p*<.001, η*_p_* ^2^ = .101) were also observed. A detailed analysis for each group was conducted and is reported below.

#### Preschool children

Significant main effects of condition were observed in the intervals of 385–435 ms (*F*(1,19) = 15.35, *p* = .001, η*_p_* ^2^ = .447), 435–485 ms (*F*(1,19) = 13.26, *p* = .002, η*_p_* ^2^ = .411), and 485–535 ms (*F*(1,19) = 14.38, *p* = .001, η*_p_* ^2^ = .431). No interactions were observed in this group.

#### School children

Significant main effects of condition were observed in the intervals of 335–385 ms (*F*(1,17) = 10.67, *p* = .005, η*_p_* ^2^ = .386), 385–435 ms (*F*(1,17) = 8.10, *p* = .011, η*_p_* ^2^ = .323), and 485–535 ms (*F*(1,17) = 7.21, *p* = .016, η*_p_* ^2^ = .298). A significant three-way interaction of condition, hemisphere, and site were observed in the intervals of 385–435 ms (*F*(2,34) = 4.89, *p* = .023, η*_p_* ^2^ = .223) and 485–535 ms (*F*(2,34) = 5.09, *p* = .017, η*_p_* ^2^ = .231). The interaction revealed a hemisphere effect at the frontal sites, that is, the deviant-standard difference was more negative at the right hemisphere electrode (F4) than the midline and left hemisphere electrodes (Fz & F3) (*p*s<.05).

#### Adult group

Significant main effects of condition were observed in the successive four time windows: 185–235 ms (*F*(1,25) = 23.59, *p*<.001, η*_p_* ^2^ = .485), 235–285 ms (*F*(1,25) = 25.56, *p*<.001, η*_p_* ^2^ = .506), 285–335 ms (*F*(1,25) = 6.20, *p* = .020, η*_p_* ^2^ = .199), and 335–385 ms (*F*(1,25) = 6.80, *p* = .015, η*_p_* ^2^ = .214). Significant interactions between condition and site were also observed in the interval of 185–235 ms, 235–285 ms, and 335–385 ms (*F*(1,25) = 4.75, 10.15, 7.89; *p* = .039, 004, 010; η*_p_* ^2^ = .160, .289, .240, respectively). A post-hoc comparison revealed that the differences between standard and deviant stimuli were more negative at the frontal electrodes than that at the central electrodes (*p*s<.005).

To summarize the results of the lexical tone contrast, the three groups exhibited a significant main effect of condition in various time windows. All of the children and adults demonstrated more negative responses to the deviant stimuli. The negative response to deviant stimuli was most evident at the frontal sites, and a clear developmental trend from late negativity in children to early negativity in the adult group was observed.

## Discussion

The main purpose of our study was using MMRs to explore the dynamic changes in Mandarin speech perception from early to middle childhood. Therefore, we used the same speech stimuli to test preschool, school-aged, and adult groups. Two sets of speech contrasts with a varying level of discriminative difficulty were used. Based on the acoustic features of the synthesized speech stimuli, we used different MMR time windows for consonant and lexical tones. The results indicated that only the adult group exhibited typical early MMN responses to both speech contrasts. The time window of the MMN response in adults was associated to the difference in acoustic features between the synthetic standard and deviant stimuli. The MMR patterns and occurring time windows in the two children groups were different from those in the adult group. This result indicated that the ability to capture specific speech features of Mandarin consonants and lexical tones in children had not yet reached the same level as it had in adults. In this study, the results of using the highly sensitive brain measures indicated the different maturation rates in discriminating specific speech features. The results of this study also provide implications of the possible indices in the change of MMRs to speech perception development.

Regarding the lexical tone pair, adults showed a typical MMN response in the time window in line with the acoustic feature manipulated. The two children groups showed negative responses in later time frames. One explanation of the late negative response is to be identified as a delayed discriminative MMN response as revealed in the adult group and the latency change suggests that children require longer processing time than adults do in discriminating sounds [25], [33]. Comparing to the two children groups, the latency of the difference between the standard and deviant stimuli occurred earlier in the school children than in the preschool group. This result confirmed that the latency of MMRs can serve as an index of developmental changes in neural speech discriminative processing as suggested in other studies [25], [29], [33]. The lexical tone pair /i2/-/i3/ in our study was comparable to the small deviance condition in two recent Mandarin Chinese studies [43], [51]. The results from the two studies showed that this acoustically similar lexical tone pair elicited p-MMRs in 6-month-old infants and children aged 4 to 6 years. Based on the statement from their studies that p-MMR reflects immature speech discrimination responses, Mandarin children younger than 6 years old are expected to show a positive response. However, our results showed no p-MMR, but the emergence of late negative response in the preschool group (mean age  = 3.4 years); therefore, the results did not correspond with the statement. The inconsistency might be explained by the difference in paradigms. In other words, the two-deviant paradigm might both create additional contextual difficulty for the same pair. This hypothesis could be verified in a direct comparison of paradigms in future studies.

Another explanation of the late negativity to lexical tone contrast in children is a LDN-like response, since the appearing time windows were in accordance to the LDN response reported in previous studies [24]–[25]. Regarding the functional significance of the speech-elicited LDN, one interpretation is that LDN is the analogue of “reorienting negativity” (RON) [52] because it follows P3a and the amplitude was observed to positively correlated with P3a [25], [53]. However, as most studies reporting LDN (including the present study), it is not necessarily preceded by P3a. The absence of orienting response eliminates the notion of reorienting as the functional significance of LDN. Zachau et al. [54] have showed that LDN could be elicited by abstract tonal patterns, and thus hypothesized that LDN reflects the process of transferring the sound pattern to the long-term memory. Based on this account, LDN in the speech condition might reflect further processing of sound structures and the establishment of internal phonological representations of speech stimuli. Therefore, the discrepancy between LDN-like response in children and early MMN in adults in this study suggests that children may not able to use the dominant pitch contour cue to discriminate Mandarin T2 and T3 as adults do and need further processing of the overall phonological structures to discriminate the two syllables. Nevertheless, it should be noted that both the delayed-MMN and LDN explanation could account for the data in this study. Therefore, the exact functional significance of the late negativity in the two children group awaits further investigation.

As for the consonant contrast, adults showed more negative responses to the deviant in early and later time frames. The early negativity appeared in the interval of 130-180 ms, which is the well-defined MMN time window by the acoustic feature differences between the standard and deviant stimuli. While this short-duration mismatch response might reflect the transient nature of the affricate-fricative pair used in this study, the interpretation of the 50-ms difference should be cautious and further replication are needed. In relation to the late negativity shown in the interval of 380-480 ms in adults, it might be taken as a LDN-like response to the consonant pairs. In a previous behavioral study, adults were observed to discriminate this contrast but did not yet reach the error-free level [40], suggesting the high level of discrimination difficulty of this contrast. Therefore, the late negativity observed in the adult group could have been caused by the high level of discrimination difficulty of this consonant pair and reflected a further processing to the phonological structures.

For the children groups, the preschool- and school-aged children in our study demonstrated positive mismatch responses between 230 to 330 ms in the consonant contrast. As for the later time frame, the results indicated that a LDN-like response resembling adults was elicited in school-aged children, but was not yet observed in the preschool group. According to previous studies, p-MMR has been observed in acoustically similar speech contrasts and have been suggested as an index of immature speech processing [43], [55]–[56]. Regarding the acoustic aspect, the variation between /t_
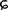
^h^_/and /_
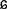
_/is in the time point of the maximal rise in amplitude during the initial 130-ms frication portion. Because the frication noise is at a high-frequency band with relatively low amplitude and is easily masked by background noise [39], the difference between the two stimuli is difficult to perceive. Thus, the observation of p-MMR in the two child groups again indicated that the neural processing of the /t_
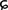
^h^_/−/_
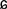
_/ contrast in children were less mature as they process the /i2/-/i3/ contrast. Compared with the results of the lexical tone contrast, the preschool group showed a LDN-like response only to the lexical tone but not in the consonant contrast. Based on our design, the consonant contrast is with higher discriminative difficulty than the lexical tone contrast. Therefore, although behavioral data from our previous study revealed no variations in discriminating /t_
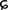
^h^_/−/_
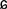
_/and /i2/-/i3/ in 4-year-old children [46], the neural response patterns found in this study provided empirical evidence reflecting the subtle developmental differences in discriminating the two speech sound pairs.

Regarding to the exact functional significance of p-MMR, it has not yet been determined. Some studies proposed that the appearance of p-MMR reflects a preliminary change-detection response and should not be identified with the P3a response observed in adults [33], [43], [51]. The overlapping of components caused by the complexity of speech signals might be one reason why studies using MMN to examine perception of phonetically similar speech contrasts generally have not reported the presence of a P3a. In the present study, we used carefully controlled synthetic stimuli containing only one varying acoustic feature. Thus, we could define the precise time window and differentiate each component elicited by the speech contrast. The p-MMR to consonant pairs in our study followed the adult MMN time window, and the timeline corresponded with the P3a mentioned in the literature. Because the P3a-like response could be obtained by using auditory stimuli in a passive condition and emerged early in 2-year-old children and exhibited a high amplitude and consistent morphology [28], [57], the functional significance of p-MMR and its relationship with the P3a component is still debatable.

Finally, for the topography domain, although Luo et al. [58] has reported that lexical tone contrasts evoked a stronger MMN in the right hemisphere than initial consonant contrasts did, our results did not indicate the same phenomenon. Based on converging data from two speech contrasts in this study, the amplitude changes in topography across various age groups (i.e., shifting from the central to frontal sites and focusing on the midline of the hemisphere aspect) may be considered an index of the developmental effect of MMRs. Nevertheless, because our study used only a small set of electrodes, the inference from the topographical changes remains to be confirmed.

In summary, the dynamic changes among MMRs in our study provide evidence supporting that children's ability to use fine-grained cues to discriminate speech contrasts in their native language is a continuing process. By using MMRs as a sensitive measure, this study also demonstrated the various developmental courses for speech features with different levels of discriminative difficulty. Based on a combination of the patterns from two speech contrasts, the results suggested that the morphology and the emergence of various ERP components (MMN, p-MMR, and LDN) may serve as indices of the neural discriminative processing of speech features. At the beginning stage, an enhancement in p-MMR might reflect the involuntary attention orienting when children fail to analyze the acoustic difference between the two speech stimuli, and thus, regard the deviant sound as the novel stimuli. When entering the advanced level, children begin to process the sound structures as reflected by the emergence of LDN and its latency change. Finally, children develop the automatic discriminatory ability of the subtle acoustic differences in their speech inventory as adults do. Our results provide empirical evidence showing the developmental process of the abilities for various speech characteristics from early to middle childhood, and indicate that the developmental patterns in MMRs are a promising tool for investigating speech perception development in future studies.
